# Effect of the Antioxidant Lipoic Acid in Aortic Phenotype in a Marfan Syndrome Mouse Model

**DOI:** 10.1155/2018/3967213

**Published:** 2018-03-25

**Authors:** Maria C. Guido, Victor Debbas, Vera M. Salemi, Elaine R. Tavares, Thayna Meirelles, Thaís L. S. Araujo, Patricia Nolasco, Julio C. A. Ferreira-Filho, Celso K. Takimura, Lygia V. Pereira, Francisco R. Laurindo

**Affiliations:** ^1^Vascular Biology Laboratory, Heart Institute (InCor), School of Medicine, University of São Paulo, São Paulo, SP, Brazil; ^2^Laboratory of Metabolism and Lipids, Heart Institute (InCor), School of Medicine, University of São Paulo, São Paulo, SP, Brazil; ^3^Heart Failure Unit, Heart Institute (InCor), School of Medicine, University of São Paulo, São Paulo, SP, Brazil; ^4^Department of Interventional Cardiology, Heart Institute (InCor), School of Medicine, University of São Paulo, São Paulo, SP, Brazil; ^5^National Laboratory for Embryonic Stem Cells (LaNCE), Department of Genetics and Evolutionary Biology, Institute of Biosciences, University of São Paulo, São Paulo, SP, Brazil

## Abstract

Marfan syndrome (MFS) cardiovascular manifestations such as aortic aneurysms and cardiomyopathy carry substantial morbidity/mortality. We investigated the effects of lipoic acid, an antioxidant, on ROS production and aortic remodeling in a MFS mgΔ^loxPneo^ mouse model. MFS and WT (wild-type) 1-month-old mice were allocated to 3 groups: untreated, treated with losartan, and treated with lipoic acid. At 6 months old, echocardiography, ROS production, and morphological analysis of aortas were performed. Aortic ROS generation in 6-month-old MFS animals was higher at advanced stages of disease in MFS. An unprecedented finding in MFS mice analyzed by OCT was the occurrence of focal inhomogeneous regions in the aortic arch, either collagen-rich extremely thickened or collagen-poor hypotrophic regions. MFS animals treated with lipoic acid showed markedly reduced ROS production and lower ERK1/2 phosphorylation; meanwhile, aortic dilation and elastic fiber breakdown were unaltered. Of note, lipoic acid treatment associated with the absence of focal inhomogeneous regions in MFS animals. Losartan reduced aortic dilation and elastic fiber breakdown despite no change in ROS generation. In conclusion, oxidant generation by itself seems neutral with respect to aneurysm progression in MFS; however, lipoic acid-mediated reduction of inhomogeneous regions may potentially associate with less anisotropy and reduced chance of dissection/rupture.

## 1. Introduction

Marfan syndrome (MFS) is a systemic multicomponent disease caused by mutations in the *Fbn1* gene, which codes for the elastic microfibril protein fibrillin-1 [1]. The MFS is caused by variable mutations, with more than 500 types described. The population incidence is rare, oscillating from 2-3 : 10.000 individual [2]. The clinical spectrum of MFS comprises ocular, musculoskeletal, and especially cardiovascular manifestations, the latter sharing a large proportion of morbidity/mortality from the disease [1]. Cardiovascular manifestations include aortic and mitral valve disease and, particularly, enlargement of the aortic root and other portions of the aorta, often leading to aneurysms, predominantly of the ascending, as-reported [3, 4], and descending thoracic and abdominal aortas. Typically, aortic dilation starts early after birth and uniformly progresses, though at variable and discontinuous rates [5], in association with elastic fiber disruption and progressive remodeling of all extracellular matrix (ECM) components [6–8]. At later stages, marked collagen deposition and disruption [9, 10] and in some cases inflammatory processes [6, 11] lead to profound vascular structural collapse. Aortic dilation is predictive of increased chance of dissection or rupture.

Several features of MFS vascular disease have been recapitulated in distinct mouse models bearing fibrillin-1 deficiencies, which have yielded translationally relevant information [12, 13]. The mgΔ^loxPneo^ is a recently described MFS mouse model, engineered by the replacement of *Fbn1* gene exons 19–24 by a neomycin-resistance expression cassette flanked by lox-P sequences. In heterozygosis, these mice present classic MFS features such as skeletal, pulmonary, and vascular abnormalities and, in particular, recapitulate the clinical heterogeneity of human MFS [14].

A converging feature of MFS pathogenesis is fibrillin-1 loss of function, which promotes disease not through a plain mechanical weakness of the ECM but through disturbed signaling, which recapitulates developmental pathways. A key mediator in this context is TGF-beta and its multiple canonical and noncanonical pathways [15]. TGF-beta neutralization in mouse models abrogates disease development [9, 16]. Moreover, TGF-beta closely interacts with angiotensin AT1 receptor signaling. Losartan markedly prevents vascular phenotype in mouse models [9], mainly through AT2 receptor-triggered inhibition of noncanonical TGF-beta signaling via ERK [17]. Of note, recent clinical trials support a relevant benefit of losartan against aortic dilation rates, although the absolute decrease seems not impressive [18] and losartan was not reportedly superior to atenolol [19]. How pathophysiological MFS signaling is orchestrated at the intra- and/or supracellular level is still elusive. A relevant candidate in this regard is the production of reactive oxygen species (ROS), which translates into organ (dys)function in many MFS-related contexts. ROS can exert beneficial or deleterious functions depending on their absolute levels, balanced against antioxidant defenses, as well as subcellular compartmentalization [20]. ROS mediate several effects of TGF-beta and angiotensin II signaling [21–23], augment endothelial dysfunction, and are produced upstream and downstream by disturbed mechanical forces associated with vascular aneurysm progression [24, 25]. ROS also play a role in ECM synthesis and degradation, for example, via matrix metalloproteinases, known to mediate MFS pathology through TGF-beta signaling [26]. The occurrence, time-course, and pathophysiological consequences associated with abnormal ROS generation in MFS are still poorly understood. Available indirect evidences include increased carbonyl-protein levels in plasma from MFS patients [27] and oxidant generation in MFS mouse aorta in correlation with endothelial dysfunction severity [28]. In the present study, we characterized the evolution of the MFS-related structural pathology in a mgΔ^loxPneo^ mouse model and the effects of the treatment with lipoic acid, an antioxidant, on ROS production and aortic disease progression.

## 2. Methods

### 2.1. Mice and Treatment Groups

A MFS mouse model mgΔ^loxPneo^ from C129/sv background was utilized in this study. These mice present a fibrillin-1 mutation, caused by the replacement of *Fbn1* gene exons 19–24 by a neomycin-resistance expression cassette flanked by lox-P sequence [14].

Mice were bred and maintained under controlled temperature and light conditions in a pathogen-free environment at the Central Animal Facility of the Medical School—University of São Paulo—and were fed a Nuvilab CR1 rodent chow (Nuvital, Colombo, Brazil) and water ad libitum.

Fifty-four MFS and 63 wild-type (WT) mice, both male and female, were used in this study. Animals were allocated to two experimental protocols:

1. Characterization of MFS mice aorta segments: in MFS and WT mice with 1, 3, and 6 months of age, ROS production, collagen and fibrillin-1 presence, wall thickness, and lumen measurements were performed in the aortic arch and thoracic and abdominal aortas. Echocardiography were also performed to assess morphometric and functional parameters of the aorta and left ventricle (LV).
2. Treatment of MFS mice with lipoic acid: to evaluate the effect of the treatment with the antioxidant lipoic acid in the aortic arch and thoracic and abdominal aortas of MFS and WT mice, animals were allocated to three groups:
  - Lipoic acid treatment: lipoic acid (0.45 g/L) was administered to mice solubilized in water. The lipoic acid dose was based on a dose-response pilot experiment where concentrations of 0.30, 0.45, and 0.60 g/L were tested, and DHE was quantified in the aorta of Marfan syndrome mice to choose the lower dose that showed reduction in oxidant generation.
  - Losartan treatment: losartan (0.6 g/L) were administered to mice solubilized in water [29, 30].
  - Untreated group: mice were kept with water ad libitum.

The animals received the treatments from 1 month until completing 6 months of age. The light-protected bottles containing lipoic acid or losartan were changed three times a week. After six months of age, echocardiography was performed and the animals were euthanized to analyze ROS production, collagen and fibrillin-1 presence, wall thickness, and lumen area. Losartan was chosen as a control group due to its known action in prevention of aortic dilation and other cardiovascular manifestations of the disease [31].

All procedures were performed in accordance with the Guidelines of the Brazilian College of Animal Research. The study protocol was approved by the Ethics Committee of the University of São Paulo Medical School Hospital (1192/09).

### 2.2. Transthoracic Echocardiography

Transthoracic echocardiography was performed using a Sequoia 512 machine (Acuson, Mountain View, CA) equipped with a 10–14 MHz linear-array transducer. Mice were anesthetized with ketamine (Cristália, Itapira, Brazil) (50 mg/kg) and xylazine (CEVA, Paulínia, Brazil) (10 mg/kg) administered by intraperitoneal injection. The heart was first imaged in the bidimensional (2-D) mode, in the parasternal long-axis view followed by the short-axis and apical 4-chamber views. Two-dimensional guided M-mode imaging was used to measure the LV end-systolic (LVESD) and end-diastolic (LVEDD) diameters, interventricular septal thickness (IVST) during diastole, and posterior wall thickness (PWT) during diastole, all in the short-axis view at the level of the papillary muscles. All measurements were done according to the American Society of Echocardiography recommendations by an experienced observer. Three representative cardiac cycles were analyzed and averaged for each measurement. LV systolic function was assessed by fractional shortening (FS) and ejection fraction (EF). In addition, aortic root diastolic diameter was measured. Doppler studies were performed to assess LV inflow and outflow; a 4-chamber view of the heart was acquired to obtain the flow parallel to the sample volume. Color Doppler flow images were obtained by centering the sampling area to the region of interest, thus making it possible to evaluate potential valvular dysfunction. Peak velocity of early (E) and late (A) diastolic filling, E/A, was obtained from the mitral inflow recordings, as previously described [32, 33].

### 2.3. Morphometry

After euthanasia, the aortas were sectioned immediately above the aortic valve and divided into three segments: the aortic arch (until the left subclavian artery), thoracic aorta (descending segment until the diaphragm), and abdominal aorta. The area analyzed as the aortic arch was the closest region to the aortic root, the thoracic aorta was the region posterior to the left subclavian artery, and the abdominal aorta was the closest to the diaphragm. One section of each aortic segment of 10 mice per group was analyzed. The fragments were formalin fixed, paraffin embedded, and cut into 5 *μ*m sections. Tissue sections were stained with hematoxylin and eosin (HE), and Masson's trichrome or Picrosirius red and Verhoeff's stain were used for morphometric studies using an image analysis system (Leica Q500 IW; Leica Imaging Systems, Cambridge, UK).

Wall thickness of aorta segments were quantified as the difference between areas delimited by external and internal elastic laminae in histological sections stained with HE under 40x magnification. Aorta collagen volume fraction (CVF) was measured in Masson's trichrome- or Picrosirius red-stained sections as the percentage of blue- or red-stained connective tissue areas per total aorta area under 400x magnification. Aortic elastic fiber disruptions were quantified by counting the number of disruptions of elastic fibers per total aorta area, in tissue sections stained with Verhoeff under 400x magnification.

### 2.4. Fibrillin-1 and Collagens I and III by Confocal Microscopy

Segments of the aortic arch and thoracic and abdominal aortas of 5-6 mice were frozen in liquid nitrogen and stored in −80°C. Later, the segments were embedded in Tissue-Tek O.C.T. Compound (Sakura Finetek, Torrance, CA) for subsequent cryostat sectioning. The area analyzed as the aortic arch was the closest region to the aortic root, the thoracic aorta was the region posterior to the left subclavian artery, and the abdominal aorta was the closest to the diaphragm. Aorta sections were fixed in acetone for 40 min, permeabilized with PBS containing 0.1% Nonidet P-40 (Sigma-Aldrich, Saint Louis, MO) for 30 min, and blocked with albumin 1% for 1 h. Primary antibodies were incubated overnight at 4°C: anti-fibrillin-1 (sc-20084, Santa Cruz, Dallas, TX), anti-collagen I (ab90395, abcam, Cambridge, MA), and anti-collagen III (ab7778, abcam, Cambridge, MA). After incubation with fluorescein-labeled secondary antibodies (1 h at 37°C), slides were mounted with PBS containing glycerol (1 : 1, *v*/*v*) and DAPI (Invitrogen, 10 *μ*g/mL). Images were obtained under 400x magnification on a Zeiss Axiovert 100M scanning confocal microscope and Axiovision software (Carl Zeiss, Jena, Germany). For expression measurements, we calculated the percentage between the positively marked area for each protein and the total tissue area.

### 2.5. Morphologic Analysis of the Aorta by Optical Coherence Tomography (OCT)

After euthanasia, mouse organs were removed from the abdominal and thoracic cavities with the exception of the aorta and heart. The aorta was retroperfused with saline solution for 10 min, followed by 10% formaldehyde for 30 min, under 100 mmHg pressure. After perfusion, the aorta was collected and kept in 10% formaldehyde. Then, it was flushed with saline solution and the optical coherence tomography (OCT) catheter introduced along the entire aortic length. Image scanning was obtained in video format for scanning film images and DICOM Medical Image format. A catheter connected to the equipment LightLab ImageWire™ OCT Imaging System (LightLab Image Inc., Westford, MA, USA) was used. The image scanning generated about 500 images of each aorta. 20 images of each aortic segment were randomly analyzed. Aortic dilation was estimated by the inner vessel area, and the wall thickness was calculated as the difference between the external and internal areas of the aortas under 100x magnification.

### 2.6. In Situ Oxidant Generation

In situ ROS microfluorotopography (which addresses total oxidant generation) of the aortic segments was performed with dihydroethidium (DHE, Invitrogen, Carlsbad, CA). Transversal aorta sections were cut in the cryostat and incubated with 5 *μ*M DHE at 37°C for 30 min. Images were obtained in a Zeiss Axiovert 100M scanning confocal microscope and Axiovision software (Carl Zeiss, Jena, Germany). Parallel reading of images was performed with identical laser acquisition settings. Control samples were preincubated with superoxide dismutase-polyethylene glycol (PEG-SOD) (500 U/mL for 7 min) to confirm the fluorescent signal as due to superoxide production [34].

ROS production was also evaluated by 5,5-dimethyl-1-pyrroline N-oxide (DMPO) (Enzo Life Sciences) *in vivo* free-radical conjugation and immunodetection of formed adducts. The nitrone spin-trap DMPO was dissolved in saline solution, and two doses were administered intraperitoneally (1 g/kg) to mice with one hour interval between them. After 3 h of monitoring, the animals were euthanized and the aortas collected and frozen embedded in Tissue-Tek O.C.T. Compound (Sakura Finetek, Torrance, CA). Aorta sections were fixed in acetone for 40 min, permeabilized with PBS containing 0.1% Nonidet P-40 (Sigma-Aldrich, Saint Louis, MO) for 30 min, and blocked with albumin 1% for 1 h. Then, the aorta was incubated with primary antibody anti-DMPO protein adduct (1 : 200) overnight at 4°C, followed by Alexa 488 secondary antibody, incubated for 2 h at 37°C. Slides were mounted with PBS containing glycerol (1 : 1, *v*/*v*) and DAPI (10 *μ*g/mL).

All images were obtained in a Zeiss Axiovert 100M scanning confocal microscope and Axiovision software (Carl Zeiss, Jena, Germany). Quantitative analysis of fluorescent images of the aortic arch and thoracic and abdominal aortas was performed with an image analysis system (Leica Q500 IW; Leica Imaging Systems, Cambridge, UK) under 400x magnification, and the percentage between the fluorescent area and the total tissue area was calculated.

### 2.7. Western Blot Analysis

Aortic arch and thoracic and abdominal aorta segments were homogenized in RIPA lysis buffer (Thermo Fisher Scientific, Waltham, MA). The proteins were size-fractionated on polyacrylamide/SDS gel. The separated proteins were transferred by electrophoresis to a nitrocellulose membrane. The membranes were blocked with 5% nonfat milk. The primary antibodies for ERK1/2 (#4696, Cell Signaling, Danvers, MA) and phosphorylated ERK1/2 (#9106, Cell Signaling, Danvers, MA) were incubated overnight, and then the blots were washed and incubated with horseradish peroxidase-conjugated secondary antibodies (Calbiochem, San Diego, CA). Bands were visualized using enhanced chemiluminescence (Amersham, GE, Fairfield, CT) and exposed and analyzed using an image analyzer (Amersham Imager 600, GE, Fairfield, CT). Results are expressed as a percentage of WT1 or WT untreated group means.

### 2.8. Statistical Analysis

Data are expressed as means ± SEM. Data were analyzed using the appropriate one-way ANOVA or repeated-measure ANOVA complemented by Bonferroni's posttest or Kruskal–Wallis with Dunn's posttest. In all analyses, *p* < 0.05 was considered statistically significant. Statistical analyses were carried out using GraphPad Prism v.5 statistical software (GraphPad Software Inc., La Jolla, CA). The data collection and analysis of echocardiographic and OCT studies were performed by a single examiner who was blinded to the animal groups and treatments.

## 3. Results

### 3.1. Characterization of MFS Mouse Aorta Segments

#### 3.1.1. Echocardiographic Structural and Functional Changes in MFS Mice at 1, 3, and 6 Months of Age

Table 1 shows the values of the echocardiographic study performed at 1, 3, and 6 months of age of MFS (MFS1, MFS3, and MFS6) and WT (WT1, WT3, and WT6) mice. All 3 MFS groups showed a greater aortic root diameter index when compared to all WT groups (*p* < 0.001). MFS1, MFS3, and MFS6 showed diastolic dysfunction, as presented by a decrease in E/A ratio (*p* < 0.001). Diastolic and systolic diameters, interventricular septum, posterior wall thickness, fractional shortening, and ejection fraction were not different between groups, meaning that there was no LV dilation, wall and interventricular septum thickening, and systolic dysfunction. Furthermore, MFS6 animals showed aortic and/or mitral regurgitation arrhythmia, bradycardia, septal hypokinesia, aortic calcification, and pulmonary hypertension.

#### 3.1.2. Aortic Remodeling in MFS Mice at 1, 3, and 6 Months of Age

The evolution of structural remodeling was assessed in the aortic arch and thoracic and abdominal aortas in MFS mice at 1, 3, and 6 months of age (Table 2). The MFS6 aortic arch and abdominal aorta wall was thicker than all other groups (*p* < 0.001). However, thoracic aorta thickness was similar in all groups.

**Figure 1:**
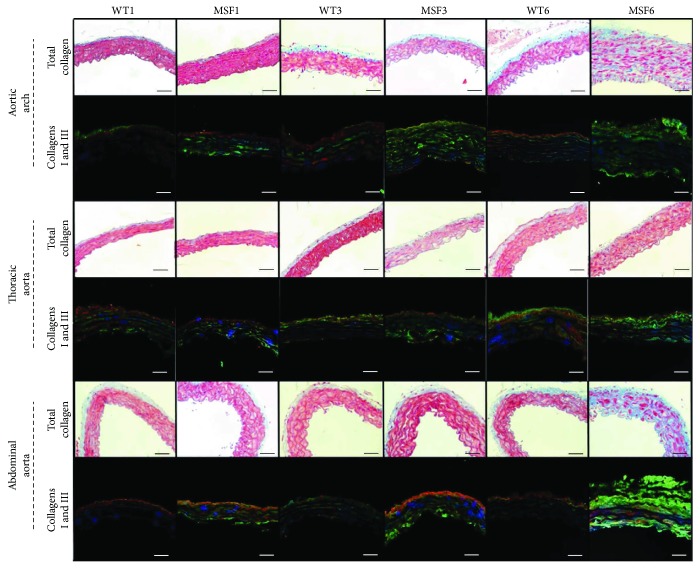
Total collagen (Masson's trichrome stain) and collagen I (green), collagen III (red), and DAPI (blue) in the aortic arch and thoracic and abdominal aortas of wild-type (WT) and Marfan syndrome (MFS) mice, at 1, 3, and 6 months of age. Magnification: 400x. Scale bars: 50 *μ*m.

Regarding collagen, MFS6 CVF was increased in the 3 segments of the aorta (*p* < 0.001), showing collagen I prevalence over collagen 3 (*p* < 0.001) (Figure 1).

**Figure 2:**
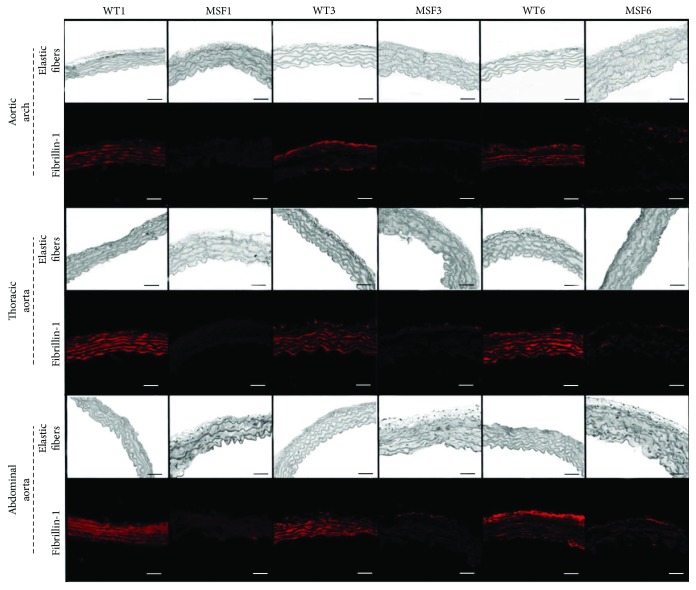
Elastic fibers and fibrillin-1 in the aortic arch and thoracic and abdominal aortas of wild-type (WT) and Marfan syndrome (MFS) mice, at 1, 3, and 6 months of age. Magnification: 400x. Scale bars: 50 *μ*m.

As expected, fibrillin-1 decreased its presence and a greater number of elastic fiber disruptions were present in the MFS groups, when compared to the WT groups (*p* < 0.001) (Figure 2).

Table 3 shows aortic dilation and wall thickness by OCT in WT and MFS mice. MF6 showed an increase in lumen and wall thickness in all aortic segments (*p* < 0.001).

**Figure 3:**
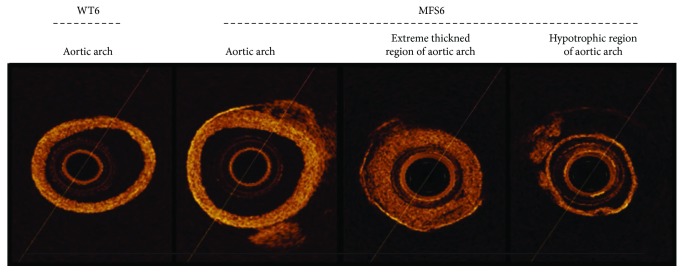
Representative optical coherence tomography (OCT) images of wild-type (WT) and Marfan syndrome (MFS) mice at 6 months of age, showing the inhomogeneous region in the aortic arch, constituted by extremely thickened and hypotrophic regions present in MFS6. Zoom: 2.7x.

Additionally, as an unprecedented finding, the analysis by OCT along the entire aortic length was able to detect inhomogeneous regions in the aortic arch in all MFS6 mice. The imaging displayed well-defined morphological characteristics that showed focal collagen-rich extremely thickened regions alternating with collagen-poor hypotrophic regions (Figure 3).

#### 3.1.3. In Situ Reactive Oxygen Species Generation in MFS Mice at 1, 3, and 6 Months of Age

**Figure 4:**
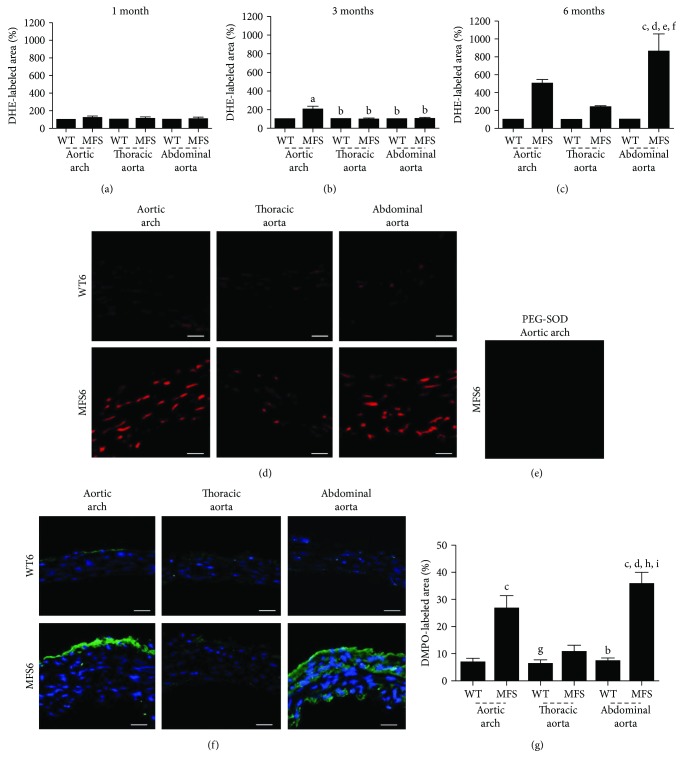
Quantitative analysis of microfluorotopography of DHE oxidation products in (a) 1-month-, (b) 3-month-, and (c) 6-month-old wild-type (WT) and Marfan syndrome (MFS) mice in the aortic arch and thoracic and abdominal aorta segments. (d) Representative photomicrographs of six-month-old wild-type (WT) and Marfan syndrome (MFS) mice, showing microfluorotopography of DHE oxidation products. Red staining indicates the fluorescence signal by DHE under 400x magnification. Bars: 50 *μ*m. (e) Sections incubated with PEG-SOD showing no reactivity. (f) Representative photomicrographs of six-month-old wild-type (WT) and Marfan syndrome (MFS) mice, showing DMPO-protein radical adduct detection in the aortic arch and thoracic and abdominal aorta segments. Green staining indicates the fluorescent signal by DMPO, and blue stain (DAPI) indicates the nuclei under 400x magnification. Bars: 50 *μ*m. (g) Quantitative analysis of DMPO-protein radical adduct detection in 6-month-old wild-type (WT) and Marfan syndrome (MFS) mice in the aortic arch and thoracic and abdominal aorta segments. ^a^*p* < 0.05 versus WT1; ^b^*p* < 0.001 versus MFS1; ^c^*p* < 0.01 versus WT1; ^d^*p* < 0.01 versus WT3; ^e^*p* < 0.01 versus MFS3; ^f^*p* < 0.01 versus WT6; ^g^*p* < 0.01 versus MFS1; ^h^*p* < 0.001 versus WT3; ^i^*p* < 0.001 versus WT6. Data expressed in mean ± SEM.

In situ ROS production measured by DHE was not different in all aorta segments of the MFS1 and WT1 groups. At 3 months, however, the MFS aortic arch presented higher ROS production when compared to all other groups (*p* < 0.01). In 6-month-old MFS mice, the abdominal aorta showed higher ROS production, when compared to all groups (*p* < 0.001). When sections were incubated with PEG-SOD, there was no reactivity, confirming that the signal obtained with DHE incubation was specifically from labeled superoxide (Figure 4).

DMPO-protein radical adduct detection corroborates the DHE results, showing higher ROS production in the MFS6 aortic arch and abdominal aorta when compared to WT aortic segments. In addition, ROS production on the abdominal aorta of MFS animals was higher than that on the thoracic aorta of MFS mice (*p* < 0.001) (Figure 4).

#### 3.1.4. pERK1/2/Total ERK1/2 Protein Expression Ratio in MFS Mice at 1, 3, and 6 Months of Age

**Figure 5:**
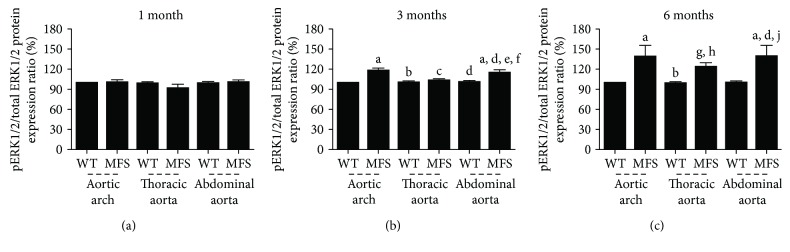
Western blot for pERK1/2/total ERK1/2. Quantitative protein expression analysis in 1-month- (a), 3-month- (b), and 6-month-old (c) wild-type (WT) and Marfan syndrome (MFS) mice untreated or treated with losartan or lipoic acid. ^a^*p* < 0.001 versus WT1; ^b^*p* < 0.001 versus MFS1; ^c^*p* < 0.01 versus MFS1; ^d^*p* < 0.001 versus WT3; ^e^*p* < 0.001 versus MFS3; ^f^*p* < 0.01 versus WT6; ^g^*p* < 0.05 versus WT1; ^h^*p* < 0.05 versus WT3; ^i^*p* < 0.05 versus MFS3; ^j^*p* < 0.001 versus WT6. Data expressed in mean ± SEM.

pERK1/2/total ERK1/2 protein expression ratio showed no difference in WT and MFS animals at 1 month of age. At 3 months, the protein expression ratio was higher in the aortic arch and abdominal aorta of MFS animals (*p* < 0.01). MFS6 mice showed higher expression in all aortic segments compared to WT aortic segments (*p* < 0.001) (Figure 5).

### 3.2. Treatment of MFS Mice with Lipoic Acid

#### 3.2.1. Echocardiographic Structural and Functional Changes in MFS Mice Treated with Lipoic Acid

Table 4 shows the values of the echocardiographic study performed in WT6 and MFS6 mice untreated or treated with losartan or lipoic acid. In the aortic root diameter index, MFS6 treated with losartan decreased the aortic root diameter index when compared with MFS6 untreated. However, MFS6 treated with lipoic acid showed a greater aortic root diameter index when compared to WT6 lipoic acid-treated group, demonstrating that the lipoic acid treatment did not affect the aortic diameter (*p* < 0.01).

Although there was no difference in LV end-systolic diameter, LV end-diastolic diameter was greater in the MFS6 lipoic acid-treated group than the WT6 and MFS6 untreated and treated with losartan groups (*P* < 0.05).

MFS6 treated with lipoic acid showed a greater interventricular septal and posterior wall thickness than WT6 and MFS6 treated with losartan. In addition, the animals WT6 and MFS6 treated with losartan showed a decrease in posterior wall thickness than WT6 and MFS6 untreated animals. Besides, WT6 animals treated with lipoic acid showed an increase in posterior wall thickness when compared to the losartan-treated groups (*p* < 0.05).

E/A ratio demonstrated that all MFS groups, regardless the treatment, showed LV diastolic dysfunction (*p* < 0.001). Moreover, LV systolic function was similar in all study groups.

#### 3.2.2. Aortic Remodeling in MFS Mice Treated with Lipoic Acid

The structural remodeling of untreated and losartan or lipoic acid treated animals was assessed in the aortic arch and thoracic and abdominal aortas (Table 5). As expected, the aortic arch thickness of the MFS6 untreated group was higher than that of the WT6 untreated group. MFS6 treated with losartan showed a lower aortic arch thickness than MFS6 untreated and MFS6 treated with lipoic acid. Thoracic aorta thickness was similar to all groups. In the abdominal aorta, MFS6 had a higher wall thickness than WT6 untreated, but the treatments with losartan or lipoic acid did not affect this parameter (*p* < 0.001).

**Figure 6:**
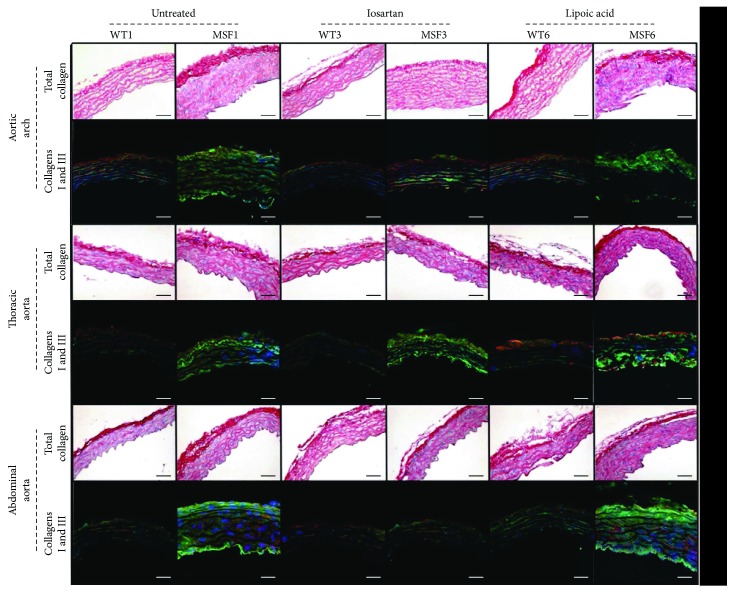
Total collagen (Picrosirius red stain), collagen I (green), collagen III (red), and DAPI (blue) in the aortic arch and thoracic and abdominal aortas of wild-type (WT) and Marfan syndrome (MFS) mice at 6 months of age untreated or treated with losartan or lipoic acid. Magnification: 400x. Scale bars: 50 *μ*m.

The CVF in the aortic arch of the MFS6 untreated group was greater than that of the WT6 untreated group. Lipoic acid did not affect CVF in the MFS6 group when compared to MSF6 untreated animals, but losartan reduced CVF in MFS6 animals when compared to MFS6 untreated and lipoic acid-treated animals. The same results were observed in the thoracic and abdominal aortas (*p* < 0.001). The collagen I presence in the aortic arch diminished in MSF6 losartan-treated animals when compared to MFS6 untreated; however, lipoic acid treatment did not change the collagen I presence. The same results were also observed in the thoracic and abdominal aortas (*p* < 0.001) (Figure 6).

**Figure 7:**
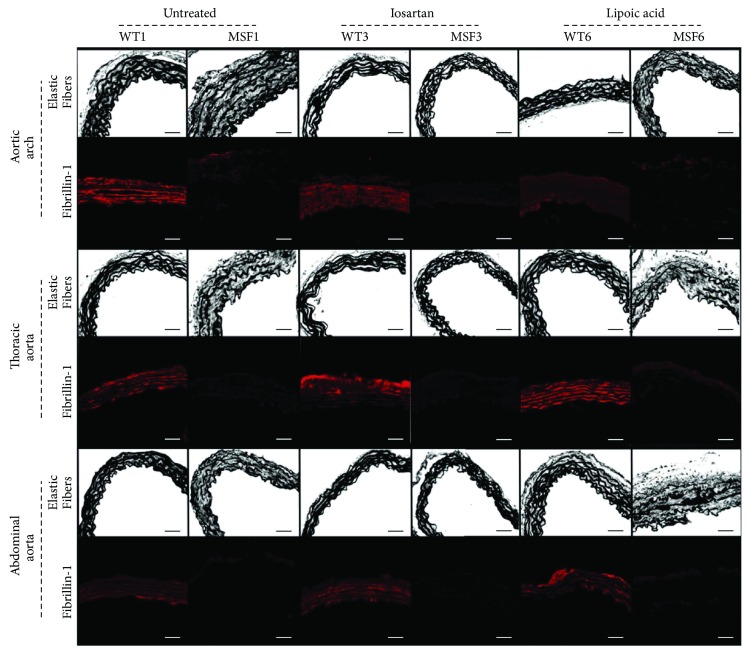
Elastic fibers and fibrillin-1 in the aortic arch and thoracic and abdominal aortas of wild-type (WT) and Marfan syndrome (MFS) mice at 6 months of age untreated or treated with losartan or lipoic acid. Magnification: 400x. Scale bars: 50 *μ*m.

Fibrillin-1 presence in the aortic arch and thoracic and abdominal aortas was decreased in all MFS6 groups compared to all WT6 groups (*p* < 0.001). However, losartan treatment reduced the number of elastic fiber disruptions in MFS6 animals compared to MF6 untreated and MFS6 lipoic acid groups (*p* < 0.001) (Figure 7).

Table 6 shows aortic dilation and wall thickness by OCT. MFS6 treatment with losartan reduced the dilation in the aortic arch and thoracic and abdominal aortas compared to MFS6 untreated and MFS6 lipoic acid-treated groups. Regarding wall thickness, the results were similar in all aortic segments (*p* < 0.001). Importantly, in MFS6 animals treated with losartan or lipoic acid, there was absence of detectable focal inhomogeneous regions in the aortic arch.

#### 3.2.3. In Situ Reactive Oxygen Species Generation in MFS Mice Treated with Lipoic Acid

**Figure 8:**
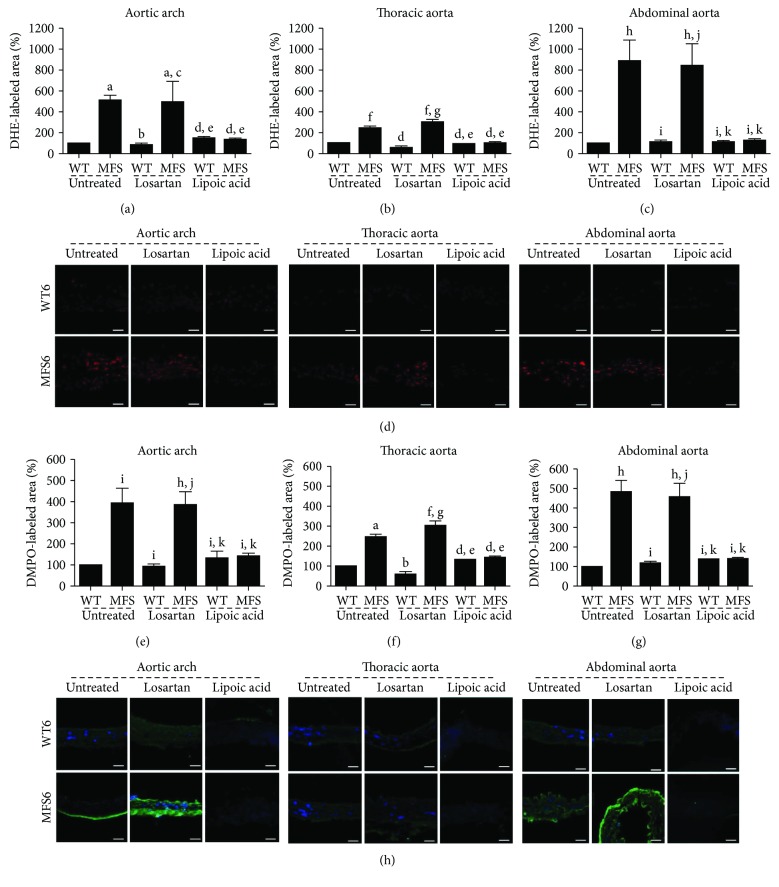
Quantitative analysis of microfluorotopography of DHE oxidation products in the (a) aortic arch, (b) thoracic aorta, and (c) abdominal aorta of wild-type (WT) and Marfan syndrome (MFS) mice untreated or treated with losartan or lipoic acid. (d) Representative photomicrographs of six-month-old wild-type (WT) and Marfan syndrome (MFS) mice, showing microfluorotopography of DHE oxidation products. Red staining indicates the fluorescence signal by DHE under 400x magnification. Bars: 50 *μ*m. Quantitative analysis of DMPO-protein radical adduct detection in the (e) aortic arch, (f) thoracic aorta, and (g) abdominal aorta of 6-month-old wild-type (WT) and Marfan syndrome (MFS) mice. (h) Representative photomicrographs of six-month-old wild-type (WT) and Marfan syndrome (MFS) mice, showing DMPO-protein radical adduct detection in the aortic arch and thoracic and abdominal aortas segments. Green staining indicates the fluorescence signal by DMPO, and blue stain (DAPI) indicates the nuclei under 400x magnification. Bars: 50 *μ*m. ^a^*p* < 0.01 versus WT untreated; ^b^*p* < 0.01 versus MFS untreated; ^c^*p* < 0.01 versus WT losartan treatment; ^d^*p* < 0.05 versus MFS untreated; ^e^*p* < 0.05 versus MFS losartan treatment; ^f^*p* < 0.05 versus WT untreated; ^g^*p* < 0.05 versus WT losartan treatment; ^h^*p* < 0.001 versus WT untreated; ^i^*p* < 0.001 versus MFS untreated; ^j^*p* < 0.001 versus WT losartan treatment; ^k^*p* < 0.001 versus MFS losartan treatment. Data expressed in mean ± SEM.

The results of DHE in MFS6 mice regarding the aortic arch and abdominal and thoracic aortas were similar. MFS6 treated with losartan was similar to MFS6 untreated animals, showing a higher ROS production than those of all WT6 groups. However, the treatment with lipoic acid markedly reduced the ROS production in MFS6 animals, displaying levels that are comparable to those of all WT6 groups (*p* < 0.001). DMPO-protein radical adduct detection corroborates the DHE results. MFS6 treated with lipoic acid substantially reduced ROS production, with similar levels to those of the WT6 groups, whereas MFS6 untreated and treated with losartan showed high levels of ROS in all aortic segments (*p* < 0.001) (Figure 8).

#### 3.2.4. pERK1/2/total ERK1/2 Protein Expression Ratio in MFS Mice Treated with Lipoic Acid

**Figure 9:**
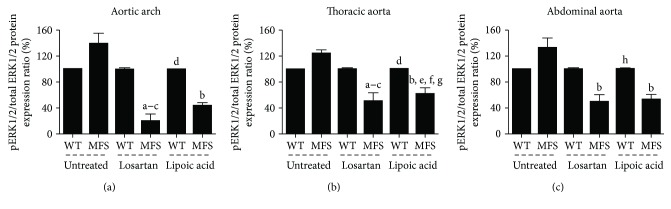
Western blot for pERK1/2/total ERK1/2. Quantitative protein expression analysis in the aortic arch (a), thoracic aorta (b), and abdominal aorta (c) of wild-type (WT) and Marfan syndrome (MFS) mice untreated or treated with losartan or lipoic acid. ^a^*p* < 0.01 versus WT losartan treatment; ^b^*p* < 0.001 versus MFS untreated; ^c^*p* < 0.01 versus WT untreated; ^d^*p* < 0.01 versus MFS losartan treatment; ^e^*p* < 0.001 versus WT untreated; ^f^*p* < 0.05 versus WT losartan treatment; ^g^*p* < 0.05 versus WT lipoic acid treatment; ^h^*p* < 0.05 versus MFS losartan treatment. Data expressed in mean ± SEM.

pERK1/2/total ERK1/2 protein expression ratio in the aortic arch and thoracic and abdominal aortas was similar. The treatment with losartan and lipoic acid strongly reduced the pERK1/2/total ERK1/2 protein expression ratio in MFS6 animals when compared to those in all WT6 groups and MFS6 untreated group (*p* < 0.001) (Figure 9).

## 4. Discussion

As the focus of the present study, we provide evidence that ROS generation at the vascular system is evident at advanced stages of disease development in MFS. In MFS6 animals, the three segments of the aorta showed high ROS production; high expression of pERK1/2; thicker aortic wall; wider lumen; greater deposition of collagen, especially collagen I; and high elastic fiber breaks. All MFS6 animals analyzed by OCT showed inhomogeneous regions in the aortic arch, constituted by extremely thickened and hypotrophic regions. To our knowledge, this is the first study to report the presence of such collagen-rich extremely thickened patches as well as collagen-poor hypotrophic regions in MFS aortic arch by OCT technique. We postulate that such focal inhomogeneous regions may enhance aortic anisotropy and favor focal events such as dissection and rupture.

On the other hand, MFS animals treated with lipoic acid showed a markedly reduced ROS production and lower expression of pERK1/2, while losartan-treated mice exhibited preserved levels of oxidant generation. Lipoic acid treatment was unassociated with changes in the progression of aortic remodeling and aneurysm formation. Losartan-treated MFS mice, as expected, markedly decreased the rates of aortic remodeling and aneurysm progression. Thus, oxidant production by itself seems neutral with respect to the progression of vascular remodeling and aneurysm in this model of MFS. However, treatment with lipoic acid prevented the appearance of focal inhomogeneous regions in the aortic arch in MFS animals, suggesting that oxidant generation may be associated with this novel process. In parallel, losartan-mediated decrease in such focal inhomogeneous regions involved ROS-independent pathways, possibly associated to the overall improvement in vascular remodeling and aneurysm progression. It is likely that both ROS-dependent and independent pathways contribute to aortic anisotropy in MFS.

The characteristic progressive dilation of the aorta is the outcome of the profusely present elastic fiber disruption in MFS. The understanding of how the genotype determines the phenotype in MFS is incomplete and, possibly, the main reason that the therapeutics is still inadequate, even when the treatments are adjusted by a strict echocardiographic control of the cardiovascular manifestations of the patient. The mainly prescribed treatments are beta-blockers, inhibitors of the angiotensin-converting enzyme inhibitors and of the angiotensin II receptors that inhibit hemodynamic stress and prevent dilation and dissection of the aorta [9, 35, 36]. Our study corroborates with these data, as losartan treatment reduced wall thickness, lumen, collagen deposition, and elastic fiber disruption in MFS aorta.

Some experimental treatments have been proposed in addition to the hemodynamic stress reduction treatment. Ramirez and Dietz [37] have shown that anti-TGF-beta antibodies can inhibit the progression of aneurysm in mice, demonstrating the role of this pathway in MFS. Another therapy proposed in experimental models was metalloproteinase inhibitors, such as doxycycline, that improve the elastic fiber integrity and VSMC contraction [38, 39].

Lipoic acid exhibits protective effects in several pathological conditions, including diabetes, hypertension, and neurodegenerative diseases. It is well accepted that lipoic acid actions in such contexts rely on its antioxidant properties, able to affect ROS-involved signaling cascades, scavenge ROS, and restore endogenous antioxidants such as reduced glutathione and supporting metal-binding activity, all resulting in reduced prooxidant burden [40, 42].

The role of redox processes in the pathophysiology of vascular alterations in MFS is not yet elucidated. Some studies show that a fibrillin-1 mutation was related to augmented ROS production, TGF-beta, and p38MAPK in MFS mice aorta [43] and that the contractility dysfunction is associated with accumulation of oxidative stress due to unbalanced superoxide enzyme protein expression [28]. A recent study showed that resveratrol inhibits aortic root dilation in MFS mice by promoting elastin integrity and improving smooth muscle cell survival [44]. Here we showed that ROS production is augmented in MFS animals. Losartan treatment was not able to reduce ROS production in MFS mice aorta; however, lipoic acid markedly reduced ROS production, to levels similar to those of WT animals without the disease.

Two signaling pathways are described to activate TGF-beta: canonic, through which TGF-beta binds to receptors and activates signaling through SMADs, and the noncanonic pathway that includes the signaling pathway of RhoA, p38MAPK, JNK, and ERK1/2. ERK1/2 is active in the aortic arch in MFS mice, and the inhibition of ERK1/2 contributed to decreased aortic remodeling and was considered an important therapeutic target [16]. Our study corroborates with this proposed pathway. Lipoic acid treatment led to significant reduction of pERK1/2 in all aortic segments. It is possible that the treatment with lipoic acid reduced ERK1/2 expression and this reduced signaling inhibits the inhomogeneous region progression in the aortic arch via reduced ROS production.

In conclusion, the treatment with the antioxidant lipoic acid markedly reduced ROS production and inhibited progression of focal inhomogeneous regions via ERK1/2-reduced signaling in the aortic arch of MFS mice. While lipoic acid was neutral with respect to aneurysm and vascular remodeling progression, it might be a possible adjuvant therapeutic agent in association to losartan regarding alternative modes of prevention of focal aortic wall inhomogeneity in Marfan syndrome.

## Figures and Tables

**Table 1:** Evolution of echocardiographic parameters in WT and MFS mice with 1, 3, and 6 months of age.

	WT1 (*n* = 10)	MFS1 (*n* = 13)	WT3 (*n* = 15)	MFS3 (*n* = 14)	WT6 (*n* = 38)	MFS6 (*n* = 27)
Ao/BW (mm/mg)	51 ± 3	73 ± 4^a^	54 ± 3^b^	67 ± 3^a,c^	47 ± 1^b,d^	63 ± 3^a,c,e^
LVEDD (mm)	3.3 ± 0.1	3.3 ± 0.1	3.2 ± 0.1	3.1 ± 0.1	3.3 ± 0.1	3.5 ± 0.1
LVESD (mm)	2.0 ± 0.1	2.0 ± 0.1	1.8 ± 0.1	1.7 ± 0.1	1.9 ± 0.1	2.0 ± 0.1
IST (mm)	0.9 ± 0.1	0.8 ± 0.1	0.6 ± 0.1	0.7 ± 0.1	0.7 ± 0.1	0.9 ± 0.1
PWT (mm)	0.7 ± 0.1	0.7 ± 0.1	0.7 ± 0.1	0.8 ± 0.1	0.7 ± 0.1	0.9 ± 0.1
FS (%)	39 ± 2	43 ± 2	44 ± 2	44 ± 2	41 ± 1	43 ± 2
EF (%)	76 ± 2	80 ± 2	80 ± 2	81 ± 2	78 ± 1	79 ± 2
E/A ratio	2.1 ± 0.1	1.1 ± 0.1^a^	1.9 ± 0.1^b^	1.2 ± 0.1^a,c^	1.9 ± 0.1^b,d^	1.2 ± 0.1^a,c,e^

WT: wild type; MFS: Marfan syndrome; 1: 1 month of age; 3: 3 months of age; 6: 6 months of age; Ao/BW: aortic root diameter index; LVEDD: left ventricular end-diastolic diameters; LVESD: left ventricular end-systolic diameters; IVST: interventricular septal thickness; PWT: posterior wall thickness; FS: fractional shortening; EF: ejection fraction; E/A: E/A waves; ^a^*p* < 0.001 versus WT1; ^b^*p* < 0.001 versus MFS1; ^c^*p* < 0.001 versus WT3; ^d^*p* < 0.001 versus MFS3; ^e^*p* < 0.001 versus WT6.

**Table 2:** Evolution of structural aorta remodeling in WT and MFS mice with 1, 3, and 6 months of age.

	WT1 (*n* = 8)	MFS1 (*n* = 6)	WT3 (*n* = 14)	MFS3 (*n* = 6)	WT6 (*n* = 7)	MFS6 (*n* = 6)
*Aortic arch*						
Thickness (mm^2^)	29 ± 1	32 ± 2	28 ± 2	36 ± 2	28 ± 1	40 ± 3^a,b,c^
Total CVF (%)	8 ± 1	8 ± 1	10 ± 1	10 ± 1	11 ± 2	23 ± 6^b–f^
Collagen I/III ratio	1.0	2.4 ± 0.6^d^	1.1 ± 0.1^g^	3.6 ± 1.2^d,e^	1.0 ± 0.1^g,h^	12.1 ± 4.1^f,i–l^
EFD (number/mm^2^)	0.10 ± 0.01	0.80 ± 0.08^i^	0.10 ± 0.02^f^	0.76 ± 0.04^i,j^	0.10 ± 0.03^f,l^	1.30 ± 0.08^i–k^
FBN1 (area %)	100.0	28 ± 9^i^	99 ± 1^f^	32 ± 6^i,j^	101 ± 1^f,l^	33 ± 5^i–k^
*Thoracic aorta*						
Thickness (mm^2^)	21 ± 1	21 ± 1	23 ± 1	22 ± 2	25 ± 1	26 ± 4
Total CVF (%)	9 ± 2	8 ± 1	14 ± 1	13 ± 2	15 ± 2	19 ± 1 ^b–d,g,m^
Collagen I/III ratio	1.0	1.9 ± 0.3^d^	0.9 ± 0.1^g^	2.2 ± 0.5^d,e^	1.0 ± 0.1^g,h^	3.1 ± 0.7^f,i–k^
EFD (number/mm^2^)	0.12 ± 0.01	0.46 ± 0.06^a^	0.11 ± 0.03^n^	0.37 ± 0.08^a,b^	0.10 ± 0.02^m,n^	0.92 ± 0.13^f,i–l^
FBN1 (area %)	100.0	25 ± 1^i^	101 ± 1^f^	21 ± 4^i,j^	100 ± 1^f,l^	48 ± 5^i–k^
*Abdominal aorta*						
Thickness (mm^2^)	15 ± 2	17 ± 1^i^	22 ± 1^f^	21 ± 2^g,i,j^	23 ± 1^f,l^	34 ± 3^i–l^
Total CVF (%)	10 ± 2	8 ± 1	11 ± 1	12 ± 2	10 ± 1	31 ± 2^f,i,j,m,o^
Collagen I/III ratio	1.0	2.0 ± 0.3^a^	1.0 ± 0.1	3.1 ± 0.9^a,b^	1.1 ± 0.1^b,n^	11.7 ± 3.2^g,i–k,n^
EFD (number/mm^2^)	0.10 ± 0.01	0.50 ± 0.10^d^	0.10 ± 0.01^g^	0.62 ± 0.14^i,j^	0.10 ± 0.01^f,l^	0.95 ± 0.19^i–k^
FBN1 (area %)	100.0	23 ± 5^i^	100 ± 1^f^	21 ± 3^i,j^	100 ± 1^f,l^	44 ± 6^d,e,o^

WT: wild type; MFS: Marfan syndrome; 1: 1 month of age; 3: 3 months of age; 6: 6 months of age; CVF: collagen volume fraction; EFD: elastic fiber disruptions; FBN 1: fibrillin-1; ^a^*p* < 0.05 versus WT1; ^b^*p* < 0.05 versus WT3; ^c^*p* < 0.05 versusWT6; ^d^*p* < 0.01 versus WT1; ^e^*p* < 0.01 versus WT3; ^f^*p* < 0.001 versus MFS1; ^g^*p* < 0.01 versus MFS1; ^h^*p* < 0.01 versusMFS3; ^i^*p* < 0.001 versus WT1; ^j^*p* < 0.001 versus WT3; ^k^*p* < 0.001 versus WT6; ^l^*p* < 0.001 versus MFS3; ^m^*p* < 0.05 versus MFS3; ^n^*p* < 0.05 versus MFS1; ^o^*p* < 0.01 versus WT6.

**Table 3:** Aortic dilation and wall thickness by optical coherence tomography analyses in WT and MFS mice with 1, 3, and 6 months of age.

	WT1	MFS1	WT3	MFS3	WT6	MFS6
	(*n* = 4)	(*n* = 4)	(*n* = 4)	(*n* = 4)	(*n* = 3)	(*n* = 5)
*Aortic arch*						
Lumen (mm^2^)	6.0 ± 0.2	6.2 ± 0.1	6.8 ± 0.2	6.8 ± 0.1	7.7 ± 0.2^a–c^	12.2 ± 1.5^c–g^
Thickness (mm)	4.8 ± 0.2	5.0 ± 0.5	5.1 ± 0.6	5.1 ± 0.8	5.2 ± 0.2	7.4 ± 0.1^c–f,h^
*Thoracic aorta*						
Lumen (mm^2^)	5.9 ± 0.2	6.1 ± 0.8	6.5 ± 0.2	6.6 ± 0.8	7.1 ± 0.2^a^	10.2 ± 0.3^c,g,i–k^
Thickness (mm)	3.8 ± 0.3	4.1 ± 0.5	3.9 ± 0.4	4.5 ± 0.8	4.0 ± 0.1	6.2 ± 0.6^c–f,h^
*Abdominal aorta*						
Lumen (mm^2^)	5.8 ± 0.2	6.1 ± 0.1	6.0 ± 0.2	6.2 ± 0.1	6.5 ± 0.2	10.9 ± 0.8^c,e,f,h,i^
Thickness (mm)	3.7 ± 0.2	4.0 ± 0.4	3.9 ± 0.4	4.2 ± 0.6	6.1 ± 0.4^k^	8.3 ± 0.1^d–g,l^

WT: wild type; MFS: Marfan syndrome; 1: 1 month of age; 3: 3 months of age; 6: 6 months of age; ^a^*p* < 0.05 versus WT1; ^b^*p* < 0.05 versus MFS1; ^c^*p* < 0.05 versus MFS3; ^d^*p* < 0.01 versusWT1; ^e^*p* < 0.01 versus MFS1; ^f^*p* < 0.01 versus WT3; ^g^*p* < 0.05 versus WT6; ^h^*p* < 0.01 versus WT6; ^i^*p* < 0.001 versus WT1; ^j^*p* < 0.001 versus MFS1; ^k^*p* < 0.001 versus MFS3; ^l^*p* < 0.05 versus MFS3.

**Table 4:** Evolution of echocardiographic parameters in WT and MFS mice with 6 months of age after losartan or lipoic acid treatments.

	Untreated		Losartan treatment		Lipoic acid treatment	
	WT6	MFS6	WT6	MFS6	WT6	MFS6
	(*n* = 38)	(*n* = 27)	(*n* = 8)	(*n* = 16)	(*n* = 7)	(*n* = 9)
Ao/BW (mm/mg)	47 ± 1	63 ± 3^a^	47 ± 1^e^	58 ± 2^f^	42 ± 2^b,c^	60 ± 2^f,g^
LVEDD (mm)	3.3 ± 0.1	3.5 ± 0.1	3.4 ± 0.1	3.2 ± 0.1	3.9 ± 0.2^k^	4.4 ± 0.2^a,e,i,j^
LVESD (mm)	1.9 ± 0.1	2.0 ± 0.1	2.2 ± 0.1	2.0 ± 0.1	2.4 ± 0.2	2.2 ± 0.1
IVST (mm)	0.7 ± 0.1	0.9 ± 0.1	0.6 ± 0.1	0.6 ± 0.1	0.8 ± 0.1	0.9 ± 0.1^c,d^
PWT (mm)	0.7 ± 0.1	0.9 ± 0.1	0.6 ± 0.1^e^	0.6 ± 0.1^e^	0.8 ± 0.1^h,k^	1.0 ± 0.1^a,i,j^
FS (%)	41 ± 1	43 ± 2	37 ± 2	38 ± 2	38 ± 3	40 ± 2
EF (%)	78 ± 1	79 ± 2	72 ± 3	75 ± 2	74 ± 3	77 ± 2
E/A ratio	1.9 ± 0.1	1.2 ± 0.1^a^	2.5 ± 0.2^b^	1.4 ± 0.1^a,i^	1.8 ± 0.1	1.3 ± 0.1^f,i^

WT: wild type; MFS: Marfan syndrome; 6: 6 months of age; Ao/BW: aortic root diameter index; LVEDD: left ventricular end-diastolic diameters; LVESD: left ventricular end- systolic diameters; IVST: interventricular septal thickness; PWT: posterior wall thickness; FS: fractional shortening; EF: ejection fraction; E/A: E/A waves; ^a^*p* < 0.001 versus untreated WT6; ^b^*p* < 0.001 versus untreated MFS6; ^c^*p* < 0.01 versus losartan treatment MFS6; ^d^*p* < 0.01 versus losartan treatment WT6; ^e^*p* < 0.01 versus untreated MFS6; ^f^*p* < 0.01 versus untreated WT6; ^g^*p* < 0.01 versus lipoic acid treatment WT6; ^h^*p* < 0.05 versus losartan treatment WT6; ^i^*p* < 0.001 versus losartan treatment WT6; ^j^*p* < 0.001 versus losartan treatment MFS6; ^k^*p* < 0.05 versus losartan treatment MFS6.

**Table 5:** Evolution of structural aorta remodeling in WT and MFS mice with 6 months of age after losartan or lipoic acid treatments.

	Untreated		Losartan treatment		Lipoic acid treatment	
	WT6	MFS6	WT6	MFS6	WT6	MFS6
	(*n* = 38)	(*n* = 27)	(*n* = 8)	(*n* = 16)	(*n* = 7)	(*n* = 9)
*Aortic arch*						
Thickness (mm^2^)	28 ± 1	40 ± 3^a^	19 ± 4^b^	23 ± 3^b^	32 ± 2	48 ± 3^c–e^
Total CVF (%)	11 ± 2	23 ± 6^a^	11 ± 1^f^	10 ± 1^b^	18 ± 2	29 ± 2^c–e,g^
Collagen I/III ratio	1.0 ± 0.1	12.1 ± 4.1^c^	1.0 ± 0.1^i^	5.2 ± 0.4^c,d^	0.8 ± 0.1^e,i^	9.2 ± 1.7^c,d,h^
EFD (number/mm^2^)	0.01 ± 0.03	1.30 ± 0.08^c^	0.01 ± 0.01^i^	0.04 ± 0.01^i^	0.02 ± 0.01^i^	1.70 ± 0.02^c–e,h^
FBN1 (area %)	101 ± 1	33 ± 5^c^	94 ± 1^i^	45 ± 5^c,d^	110 ± 2^e,i^	39 ± 2^c,d,h^
*Thoracic aorta*						
Thickness (mm^2^)	25 ± 1	26 ± 4	25 ± 2	20 ± 1	27 ± 1	33 ± 1
Total CVF (%)	15 ± 2	19 ± 1	12 ± 3	20 ± 3	16 ± 1	21 ± 6^c–e,j^
Collagen I/III ratio	1.0 ± 0.1	3.1 ± 0.7^c^	1.3 ± 0.2^i^	1.5 ± 0.1^c,d^	1.5 ± 0.1^e,i^	3.1 ± 0.2^c,d,f,h^
EFD (number/mm^2^)	0.10 ± 0.02	0.92 ± 0.13^c^	0.11 ± 0.01^i^	0.38 ± 0.01^i^	0.13 ± 0.02^i^	1.01 ± 0.25^c–e,h^
FBN1 (area %)	100 ± 1	48 ± 5^c^	90 ± 1^i^	57 ± 3^c,d^	101 ± 1^e,i^	39 ± 3^c,d,h^
*Abdominal aorta*						
Thickness (mm^2^)	23 ± 1	34 ± 3^k^	27 ± 6^f^	20 ± 2	23 ± 3	34 ± 4
Total CVF (%)	10 ± 1	31 ± 2^a^	10 ± 1^f^	12 ± 2^f^	11 ± 2	32 ± 3^c,d,f,h,i^
Collagen I/III ratio	1.1 ± 0.1	11.7 ± 3.2^c^	0.8 ± 0.1^i^	1.1 ± 0.1^c,d^	1.0 ± 0.3^e,i^	10.2 ± 0.1^c,d,f,h^
EFD (number/mm^2^)	0.10 ± 0.01	0.95 ± 0.19^c^	0.11 ± 0.01^b^	0.50 ± 0.10^i^	0.12 ± 0.2	1.40 ± 0.50^c–e,h^
FBN1 (area %)	100 ± 2	44 ± 6^c^	87 ± 1^i^	31 ± 6^c,d^	98 ± 4^e,i^	38 ± 3^c,d,h^

WT: wild type; MFS: Marfan syndrome; 6: 6 months of age; CVF: collagen volume fraction; EFD: elastic fiber disruptions; FBN 1: fibrillin-1; ^a^*p* < 0.01 versus untreated WT6; ^b^*p* < 0.01 versus untreated MFS6; ^c^*p* < 0.001 versus untreated WT6; ^d^*p* < 0.001 versus losartan treatment WT6; ^e^*p* < 0.001 versus losartan treatment MFS6; ^f^*p* < 0.05 versus untreated MFS6; ^g^*p* < 0.01 versus lipoic acid treatment WT6; ^h^*p* < 0.001 versus lipoic acid treatment WT6; ^i^*p* < 0.001 versus untreated MFS6; ^j^*p* < 0.05 versus lipoic acid treatment WT6; ^k^*p* < 0.05 versus untreated WT6.

**Table 6:** Aortic dilation and wall thickness by optical coherence tomography analyses in WT and MFS mice with 6 months of age after losartan or lipoic acid treatments.

	Untreated		Losartan treatment		Lipoic acid treatment	
	WT6	MFS6	WT6	MFS6	WT6	MFS6
	(*n* = 3)	(*n* = 4)	(*n* = 3)	(*n* = 4)	(*n* = 4)	(*n* = 5)
*Aortic arch*						
Lumen (mm^2^)	7.7 ± 0.2	12.2 ± 1.5	8.2 ± 0.9	6.3 ± 0.4	9.3 ± 0.6	16.0 ± 1.3^a–d^
Thickness (mm)	5.2 ± 0.2	7.4 ± 0.1	5.1 ± 0.4	4.6 ± 0.4	5.7 ± 1.2	10.4 ± 1.2^e–g^
*Thoracic aorta*						
Lumen (mm^2^)	7.1 ± 0.2	10.2 ± 0.3	7.5 ± 0.9	5.6 ± 0.5	8.1 ± 1.3	12.8 ± 1.2^e,g^
Thickness (mm)	4.0 ± 0.1	6.2 ± 0.6^e^	3.8 ± 0.2^h^	4.0 ± 0.2^h^	5.4 ± 0.4	8.4 ± 0.3^h–l^
*Abdominal aorta*						
Lumen (mm^2^)	6.5 ± 0.2	10.9 ± 0.8^a^	6.5 ± 0.9^m^	4.3 ± 0.1^n^	7.3 ± 0.3^g,h^	10.6 ± 0.1^b,i,k,l^
Thickness (mm)	6.1 ± 0.4	8.3 ± 0.1^a^	6.5 ± 0.1^h^	6.3 ± 0.1^m^	6.6 ± 0.4^m^	9.8 ± 0.1^i–k,o^

WT: wild type; MFS: Marfan syndrome; 6: 6 months of age; ^a^*p* < 0.01 versus untreated WT6; ^b^*p* < 0.01 versus losartan treatment WT6; ^c^*p* < 0.01 versus losartan treatment MFS6; ^d^*p* < 0.05 versus lipoic acid treatment WT6; ^e^*p* < 0.05 versus untreated WT6; ^f^*p* < 0.05 versus losartan treatment WT6; ^g^*p* < 0.05 versus losartan treatment MFS6; ^h^*p* < 0.05 versus untreated MFS6; ^i^*p* < 0.001 versus untreated WT6; ^j^*p* < 0.001 versus losartan treatment WT6; ^k^*p* < 0.001 versus losartan treatment MFS6; ^l^*p* < 0.01 versus lipoic acid treatment WT6; ^m^*p* < 0.01 versus untreated MFS6; ^n^*p* < 0.001 versus untreated MFS6; ^o^*p* < 0.001 versus lipoic acid treatment WT6.
